# Prognostic Factors in The Development of Permanent Renal Injury in Patients Undergoing Cardiac Surgery

**DOI:** 10.1186/2197-425X-3-S1-A14

**Published:** 2015-10-01

**Authors:** C Rodriguez Mejias, MR Diaz Contreras, L Navarro Guillamon, E Aguayo de Hoyos, A Reina Toral

**Affiliations:** Granada University, ICU Complejo Hospitalario, Granada, Spain

## Introduction

Renal injury is a frequent and serious complication in patients undergoing cardiac surgery. This injury usually recover completely. However, according to recently published data, a significant number of patients with this lesion remain at hospital discharge.

## Objective

To analyze patients undergoing cardiac surgery in our hospital to determine whether there prognostic factors for the development of permanent kidney damage.

## Methods

We use data base Andalusian registry of cardiac surgery in the comprehensive care plan to heart disease, referring to patients operated in our hospital and admitted to our intensive care unit of the” Hospital Virgen de las Nieves” of Granada, between June 2008 until the end of 2014.

## Results

A total of 2,443 patients underwent cardiac surgery and discharged from hospital alive. of these 1,497 had prior to surgery normal renal function (defined by plasma creatinine ≤ 1.2 mg). a total of 149 were discharged with plasma creatinine levels that exceeded 0.3 mg baseline and / or an increase of 50% of these values, regardless of what happened during their hospital stay.

The main baseline characteristics of patients with and without renal injury were (table 1)

By adjusting a model of binary logistic regression (table 2) with the variables age, diagnostic group, NYHA class, character surgery, COPD (cronic obstructive pulmonary disease), Diabetes, ejection fraction and EuroSCORE is obtained that the variables that are significantly associated with the development of renal injury are age (most significant), COPD and NYHA class.

**Table 1:** Table 1

	No (1678)		Yes (149)		
	Media	sd	Media	sd	p
Age	61,44	13,59	67,30	10,64	,000
Preoperative creatinine	,89	,17	,86	,19	,05
Ejection fraction	59,25	10,52	57,43	11,07	,04
CPB time	112,37	45,91	108,14	42,94	,3
Aortic clamp time	85,61	38,33	86,09	38,47	,88
Discharge creatinine	,80	,20	1,49	,77	,000
EuroSCORE Log	6,94	7,85	9,55	11,38	,000

**Table 2:** Table 2

	No n	No %	Yes n	Yes %	p
Diabetes	415	25,00	56	38,10	,001
COPD	151	9,10	24	16,30	,008
NYHA:I	473	28,20	29	19,50	,002
II	700	41,80	53	35,60	
III	439	26,20	57	38,30	
IV	64	3,80	10	6,70	
Type: Emergent	32	1,90	5	3,40	,25
Scheduled	1448	86,30	122	81,90	
Urgent	198	11,80	22	14,80	

## Conclusions

Age, having COPD or NYHA class to undergo cardiac surgery, are prognostic factors for the development of permanent renal injury. These factors should be considered in risk stratification prior to surgery.

